# Class 1 Integrons and the Antiseptic Resistance Gene (*qacEΔ1*) in Municipal and Swine Slaughterhouse Wastewater Treatment Plants and Wastewater—Associated Methicillin-Resistant *Staphylococcus aureus*

**DOI:** 10.3390/ijerph120606249

**Published:** 2015-06-02

**Authors:** Min Tao Wan, Chin Cheng Chou

**Affiliations:** 1 School of Veterinary Medicine, National Taiwan University, No. 1, Section 4, Roosevelt Road, Taipei 106, Taiwan; E-Mail: d99629002@ntu.edu.tw; 2 Biodiversity Research Center, Academia Sinica, 128 Academia Road, Section 2, Taipei 115, Taiwan

**Keywords:** methicillin-resistant *Staphylococcus aureus*, class 1 integrons and antiseptic resistance genes, wastewater

## Abstract

Class 1 integrons are mobile gene elements (MGEs) containing *qacEΔ1* that are resistant to quaternary ammonium compound (QAC) disinfectants. This study compared the abundances of class 1 integrons and antiseptic resistance genes in municipal (M) and swine slaughterhouse (S) wastewater treatment plants (WWTPs) and investigated the presence of class 1 integrons and antiseptic resistance genes in methicillin-resistant *Staphylococcus aureus* (MRSA) isolated from wastewater samples. The abundances of *intI1* and *qacEΔ1* genes in 96 wastewater samples were quantified using real-time quantitative polymerase chain reaction (real-time qPCR), and 113 MRSA isolates recovered from the wastewater samples were detected class 1 integrons and linked antiseptic resistance genes (*qacEΔ1*), and minimum inhibitory concentrations (MICs) for QAC antiseptics. The *intI1* and *qacEΔ1* genes were detected in all the wastewater samples, and they were more abundant in S-WWTP samples than in M-WWTP samples. A higher percentage of MRSA isolates carried *qacEΔ1* in MRSA from swine wastewater samples (62.8%) than in municipal MRSA (3.7%). All the MRSA isolates showed high MICs for antiseptic agents. This study provides important evidence regarding the abundances of *intI1* and *qacEΔ1* genes in municipal and swine slaughterhouse wastewater, and antiseptic-resistant MRSA strains were detected in swine slaughterhouse wastewater.

## 1. Introduction

Antibiotic-resistant bacteria (ARB) have been found in aquatic environments, including surface water and groundwater [1,2]. Wastewater has been reported to be a significant hotspot for the development and dissemination of ARB and antibiotic-resistant genes (ARGs) through horizontal gene transfer [3]. Methicillin-resistant *Staphylococcus aureus* (MRSA) is one of the ARB reported in human and animal hospitals, and it is the cause of many serious infections such as osteomyelitis, endocarditis, meningitis, pneumonia, and bacteraemia [4,5]. Several studies have documented the presence of MRSA in various environmental water bodies including wastewater [3,6,7]; therefore, MRSA is considered to be an emerging contaminant in water environments [3].

Integrons are mobile DNA elements that are frequently associated with antibiotic resistance [8]. Integrons consist of two conserved segments (CSs) usually separated by a variable region that includes mobile cassettes with antibiotic resistance genes [8]. In class 1 integrons, the 5’-CS harbors the class 1 integrase gene (*intI1*) and an attachment site (*attI*) that can capture and express resistant genes [8,9]. The 3’-CS contains *qacEΔ1* and *sul1*, which encode resistance to disinfectants of quaternary ammonium compounds (QACs) and sulfonamides, respectively [8,9]. In contrast to major public concern caused by antibiotic resistance in staphylococci, resistance to antiseptics has received attention [10]. The antiseptic resistance is attributable to carriage of plasmids harboring genes that increase tolerance to many agents including QACs [10], which are often used as major ingredients in environmental disinfectants.

The occurrence of class 1 integrons in nosocomial MRSA strains was first documented in 2007 [11]. The presence of class 1 integrons in livestock environments has likewise been previously reported [12]. For example, class 1 integrons are abundant in various water environments, including wastewater [13,14], and may be a major environmental contaminant and cause public health problems [15]. Resistance to QACs was developed in Gram-negative bacteria, such as in Enterobacteriaceae (e.g., *Citrobacter freundii* and *Enterobacter cloacae*) and *Pseudomonas aeruginosa* [16]. There are reports on *Staphylococcus aureus* harboring QAC-resistant genes, from human (e.g., *qac*A/B) and animals (e.g., *qac*G, *qac*H, and *qac*J), which demonstrate decreased susceptibility to disinfectants such as benzalkonium chloride (BKC) [17,18,19,20]. However, the knowledge on the coexistence and the abundance of class 1 integrons and linked *qacEΔ1* genes in the wastewater environment is poor, and little is known regarding the occurrence of class 1 integrons and *qacEΔ1* genes in MRSA in wastewater.

This study aimed (1) to quantify and to compare the abundances of class 1 integrons and the *qacEΔ1* genes in wastewater; and (2) to investigate the presence of class 1 integrons and *qacEΔ1* genes in MRSA strains isolated from different wastewater sources. Wastewater samples from one municipal wastewater treatment plant (M-WWTP) and one swine slaughterhouse wastewater treatment plant (S-WWTP) were analyzed by real-time quantitative polymerase chain reaction (real-time qPCR). The results provide quantifications and distributions of class 1 integrons and *qacEΔ1* genes in the spread of different wastewater sources and wastewater-associated MRSA.

## 2. Materials and Methods

### 2.1. Wastewater Treatment and Sampling

**Figure 1:**
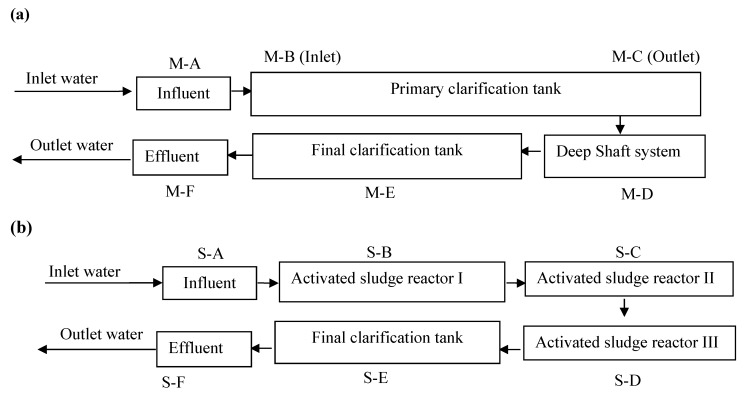
(**a**) Schematic drawing of the process at the municipal wastewater treatment plant, M-WWTP, and (**b**) the swine slaughterhouse wastewater treatment plant, S-WWTP [21].

The municipal WWTP (M-WWTP) from which samples were taken for this study is one of the largest secondary treatment plants in Taiwan, receiving Taipei municipal wastewater with an average daily flow of 500,000 m^3^. The system is designed for primary treatment (*i.e.*, the physical removal of solids), secondary treatment (*i.e.*, biological processing), final clarification, and the chlorination treatment of outgoing water. Wastewater samples taken at sampling site M-A were the incoming influent wastewater at the M-WWTP; samples taken at sampling site M-B and M-C were wastewater before and after solid removal during primary treatment, the hydraulic retention time (HRT) between both is 1.1 h; samples taken at sampling site M–D were after secondary treatment for activated sludge process, the HRT is 4.8 h; samples M–E were taken from final clarification site, the HRT is 3.8 h, and samples M-F were the effluent water taken after chlorination treatment with HRT of 25.5 min. The S-WWTP from which samples were taken for this study is the treatment plant for the second largest swine auction market with pigs ready for slaughter in Taiwan, receiving wastewater with an average daily flow of 800 m^3^. The system is designed for primary treatment (solid/liquid separation), secondary treatment (activated sludge processing), final clarification, and the treatment of outgoing water (Figure 1). The incoming wastewater at the S-WWTP includes wastewater from the slaughterhouse of swine origin only. The processes completed at the S-WWTP include primary treatment (solid/liquid separation, sampling site S–A, HRT 1 h), activated sludge process I-III (sampling site S–B to S–D, HRT 6 h), final clarification (sampling site S–E, HRT 1 h), and the treatment of outgoing water (sampling site S–F). A total of 96 wastewater samples (24 from the M-WWTP and 72 from the S-WWTP) were collected between 2010 and 2011. Each sample consisted of 250 mL of wastewater placed in a sterile 500 mL Whirl-Pak bag and stored at 4 °C during transportation, which lasted less than 3 h [20].

### 2.2. Concentration and Total DNA Extraction

Wastewater concentration and total DNA extraction were performed according to the process described in our previous protocol [21]. Briefly, aliquots of 100 mL of wastewater were centrifuged (8000 *g* for 30 min) and the pellets were immediately extracted using the QIAamp^®^ DNA Stool Mini kit (Qiagen GmbH, Hilden, Germany) according to the manufacturer’s protocol. The extracted DNA was then stored at −80 °C prior to use.

### 2.3. Real-Time qPCR Standards

The real-time qPCR standard plasmids of *intI1* and *qacEΔ1* were constructed as described in our previous study [21]. Briefly, the target fragments of the *intI1* (473-bp) and *qacEΔ1* (225-bp) were first amplified by conventional PCR according to the designed primers of Hardwick *et al.* [13] and Sandvang *et al.* [22], respectively. Thereafter, PCR products were purified and cloned into a pGEM-T Easy vector system (Promega, Madison, WI, USA). Colonies containing the vector were grown in LB broth for 12–16 h at 37 °C, and recombinant plasmid DNA was extracted using quick plasmid preparation (Mini Plus™ Plasmid DNA Extraction System, Viogene, New Taipei City, Taiwan). After standard plasmid DNA extraction and purification, the plasmid was linearized using *Xmn*I restriction enzyme (Promega) and complete digestion was confirmed based on the banding pattern observed after agarose gel electrophoresis. All the linearized plasmid was then purified using the QIAquick PCR purification kit (Qiagen GmbH). The number of copies of the targeted gene was calculated using the formula reported by Colomer-Lluch, *et al.* [23]. Ten-fold serial dilution of the standard plasmid, ranging from 10^3^ to 10^11^ copies μL^−1^, was performed to prepare the standard curves for the real-time qPCR of the *intI1* and *qacEΔ1*. All standard plasmids were stored at −80 °C prior to use.

### 2.4. Real-Time qPCR

Real-time qPCR assay was performed using the florescent quantitative Line gene 9600 system (Hangzhou Bioer Technology, Zehjiang, China) with a total of 20 μL of Smart Quant Green Master Mix with dUTP Low ROX mixture (Protech Technology, Taipei, Taiwan) as described previously [14]. All reactions were conducted in triplicate. The criteria of acceptability for each real-time qPCR assay were described in our previous study [20] and were defined as a correlation coefficient value (R^2^) > 0.98 and an efficiency percentage between 90% and 110% (slope: −3.6 and −3.10). The reproducibility of real-time qPCR was evaluated by the coefficient of variation (CV%) in three threshold cycle (Ct) values. A CV < 10% was acceptable. Gene quantities of *intI1* and *qacEΔ1* were normalized gene copies µg^−1^ of total DNA.

### 2.5. MRSA Isolation, Identification, Class 1 Integrons and QAC Genes Detection

MRSA isolation from slaughterhouse wastewater was performed as described in our previous study [21]. In brief, 100 mL of wastewater was centrifuged at 8000 *g* for 30 min. The resulting pellet was then resuspended in 1 mL of the original wastewater. This 1-mL suspension was then placed into 3 mL tryptic soy broth (TSB) medium with 6.5% NaCl (Difco, Sparks, MD, USA) for enrichment at 37 °C for 24 h. A loopful of the TSB was then streaked onto selective CHROMagar™ MRSA (CHROMagar™ Microbiology, Paris, France) and incubated at 37 °C for 24 h. The suspected MRSA colonies (mauve or pink color) were inoculated into 5 mL brain heart infusion broth (Difco) for 16 h at 37 °C and then streaked onto Baird-Parker agar (Difco) with 5% egg yolk tellurite-supplements for 48 h at 37 °C. The colonies were tested by 6 μg mL^−1^ of oxacillin Mueller–Hinton agar (Difco) with 4% NaCl and 4 μg mL^−1^ of cefoxitin (broth micro-dilution) for MRSA phenotypic screening. The MRSA colonies were confirmed using PCR to identify the *nuc* and *mecA* genes as described previously [24,25]. Plasmid DNA from MRSA isolates was extracted using Mini Plus™ Plasmid DNA Extraction System (Viogene, New Taipei City, Taiwan). The MRSA isolates were stored at −80 °C prior to use. The detection of *qacEΔ1* (225-bp) and *intI1* (473-bp) genes followed the protocols described by Sandvang, *et al.*, and [22], Hardwick *et al.* [13]. The details of the primer pairs used to amplify each gene in this study are shown in Table 1.

**Table 1:** Primers used in this study.

Target Genes	Primers	Sequence (5’–3’)	Amplicons (bp)	Annealing Temperature (°C)
*intI1*	HS463a HS464	CTGGATTTCGATCACGGCACG ACATGCGTGTAAATCATCGTCG	473	60
*qacEΔ1*	*qacEΔ1F* *qacEΔ1B*	ATCGCAATAGTTGGCGAAGT CAAGCTTTTGCCCATGAAGC	225	60
*nuc*	*nuc*-1 *nuc*-2	GCGATTGATGGTGATACGGTI AGCCAAGCCTTGACGAACTAAAGC	280	55
*mec*A	Forward Reverse	CTCAGGTACTGCTATCCACC CACTTGGTATATCTTCACC	448	42

### 2.6. Minimum Inhibitory Concentration of Antiseptics

The minimal inhibitory concentrations (MICs) of the antiseptics (benzethonium chloride and benzalkonium chloride) of the MRSA isolates were determined using broth micro-dilution according to the CLSI M31-A3 guidelines [26].

### 2.7. Statistical Analysis

STATISTICA 8.0 (StatSoft Inc., Tulsa, OK, USA) was used for the statistical analyses. The correlations between the class 1 integrons and *qacEΔ1* were computed using the Spearman test. The significance of the correlations was computed using Kendall’s tau correlation analysis. The abundance of *intI1* and *qacEΔ1* in the influent and effluent from M-WWTP and S-WWTP wastewater was compared using the Mann-Whitney U test. A *P* value <0.05 was considered statistically significant.

## 3. Results

### 3.1. The Abundances of Inti1 and QacEΔ1 in Wastewater

**Figure 2:**
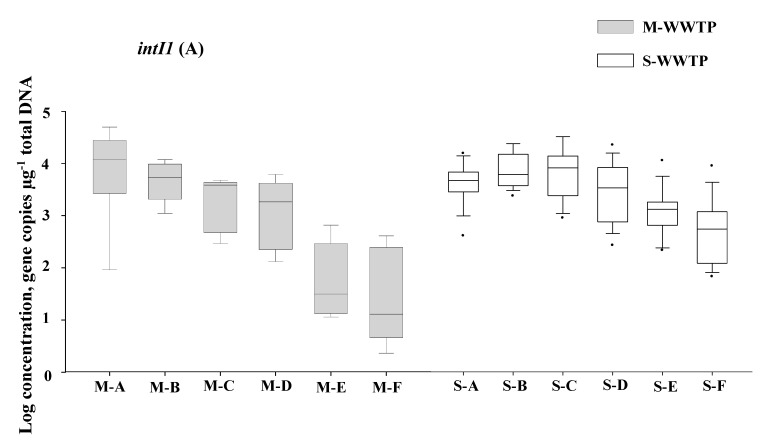

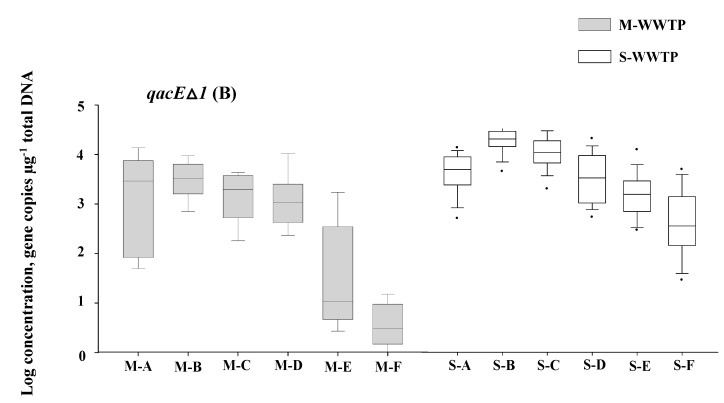
Abundance of *intI1* (A) and *qacEΔ1* (B) determined with the SYBR Green real-time qPCR. M-WWTP: municipal wastewater treatment plant. S-WWTP: swine slaughterhouse wastewater treatment plant. The following sampling sites are shown: M–A: influent, M–B: inlet of the primary clarification tank, M-C: outlet of the primary clarification tank, M–D: deep shaft system, M–E: secondary clarification tank, M–F: effluent; S–A: influent, S–B to S–D: activated sludge reactor I-III, S–E: final clarification tank, S–F: effluent. Within the box and whiskers plot, the bottom and top of the box plot represent 25th, median (black bar) and 75th percentiles. The whiskers indicate the 5th and 95th percentiles. Black dots represent outlier (s).

All 96 of the wastewater samples taken tested positive for *intI1* and *qacEΔ1*. The levels of *intI1* (1.8–5.4 log_10_; 7 × 10^1^ to 2.5 × 10^5^ gene copies μg^−1^ of total DNA) and *qacEΔ1* (1.5–5.4 log_10_; 3.2 × 10^1^ to 2.5 × 10^5^ gene copies μg^−1^ of total DNA) in S-WWTP wastewater were higher than those (*intI1*: 0.3 to 4.7 log_10_; 2.2 to 5.5 × 10^4^ gene copies μg^−1^ of total DNA, and *qacEΔ1*: 0.26 to 4.1 log_10_; 1.8 to 1.4 × 10^4^ gene copies μg^−1^ of total DNA) in the M-WWTP samples (Figure 2).

Following the wastewater treatment processes, changes of the *intI1* and *qacEΔ1* levels in the M-WWTP samples (4.1 to 1.1 log_10_ median units and 3.5 to 0.5 log_10_ median units, respectively; *p* < 0.05) were higher than those (4.1 to 3.0 log_10_ median units and 4.1 to 2.8 log_10_ median units, respectively; *p* < 0.05) in the S-WWTP samples. The most significant reductions of the *intI1* and *qacEΔ1* levels were observed after the clarification process for both treatment systems (*p* < 0.05). Thus, the levels of *qacEΔ1* and *intI1* were also higher in the effluent of the S-WWTP than they were in the effluent of the M-WWTP (*p* < 0.05). A positive correlation was observed between the number of gene copies of *intI1* and *qacEΔ1* in both the M-WWTP and S-WWTP samples (R = 0.87, *p* < 0.05; data not shown).

### 3.2. Detection of IntI1 and QacEΔ1 in MRSA Isolates and MICs Testing for QacEΔ1-Positive MRSA

Gene *intI1* was not detected either in the genomic DNA or in the plasmid DNA of 113 MRSA (86 isolates from S-WWTP and 27 isolates from M-WWTP). Overall, fifty-five MRSA isolates (48.7%, 55/113) harbored *qacEΔ1* genes. Fifty-four MRSA isolates from the S-WWTP (62.8%, 54/86) were identified carrying *qacEΔ1* genes. In contrast, only 1 isolate from M-WWTP (3.7%, 1/27) was carried the *qacEΔ1* genes. Isolates harboring *qacEΔ1* had observed MIC ranges of benzethonium chloride and benzalkonium chloride of 1–8 μg mL^−1^ and 2–256 μg mL^−1^, with MIC_50_ and MIC_90_ values of 4 and 8 μg mL^−1^ and 4 and 16 μg mL^−1^, respectively.

## 4. Discussion

In the present study, we discovered different abundances of class 1 integrons and linked antiseptic- resistance genes in wastewater samples from a municipal origin and a swine slaughterhouse, respectively. Firstly, class 1 integrons (*intI1*) and QAC-resistant (*qacEΔ1*) genes were found at stable levels in sample from both types of WWTPs. Secondly, even after treatment, different levels of class 1 integrons and QAC-resistant genes were discharged into the effluent of municipal and swine slaughterhouse wastewaters. Lastly, MRSA isolates from the wastewater of the S-WWTP had high level of antiseptic-resistance genes and also high antiseptic MIC values.

The results of the detection of *intI1* over time in the M-WWTP samples are in agreement with previous studies that integrons carrying various ARGs are common in water environments, such as waters from municipal sewage treatment plants [14,27,28]. The *intI1* and *qacEΔ1* genes were also detected in 100% of the S-WWTP samples. One possible explanation for the high detection of *intI1* and *qacEΔ1* in S-WWTP isolates is the high prevalence of Gram-negative bacteria, such as *Salmonella* and *Escherichia coli*, carrying class 1 integron genes in swine in Taiwan [29]. The quantification of *intI1* in both of M-WWTP and S-WWTP appears to be more abundant than *qacEΔ1*. This may due to class 1 integrons can have different structures that include types with no 3’-CS [30]. Our results showed a positive correlation between the *intI1* and *qacEΔ1* genes. The findings are consistent with Gaze, *et al.* [31] report that class 1 integrons have the same increasing frequencies with the increasing frequencies of QAC resistances. These indicate an important relationship between *qac* genes and class 1 integrons [32].

The effluents of M-WWTP and S-WWTP contained more than 5 log_10_ gene copies μg^1^ of total DNA of *intI1* and *qacEΔ1* after treatment, and the quantities of the selected genes in the effluent of the S-WWTP were higher than those in the effluent of the M-WWTP by one order of magnitude. These results suggested that the continuous discharge of municipal wastewater and swine slaughterhouse wastewater may release *intI1* and *qacEΔ1* into natural water environments, implying that M-WWTP and S-WWTP wastewaters may cause the spread of harmful genetic determinants into natural environments and act as hotspots for the transformation of normal bacterial flora in environmental water to ARB through horizontal gene transfer.

By comparing the effluent to the influent of both types of wastewater, the treatment processes were found to significantly influence the abundances of *intI1* and *qacEΔ1*. The *qacEΔ1* and *intI1* levels in the effluent of the M-WWTP samples were decreased by a greater amount than they were in the effluent of the S-WWTP samples. These differences may be due to the relatively advanced treatment systems used at the M-WWTP compared to those used at the S-WWTP, such that the secondary clarification processes of the M-WWTP had higher removal rates for resistant genes [14]. However, most of the selected genes were still present even after treatment in the effluent of both types of samples. This implies that standard biological treatment processes are not good enough to remove those resistant genes in wastewater, and better advanced treatment processes are recommended.

More than 60% of Taiwan livestock associated-MRSA ST9 (Taiwan LA-MRSA ST9) isolates from swine slaughterhouse wastewater in the present study harbored *qacEΔ1* which is higher than the percentage (43.8%) previously found for human MRSA isolates with *qac*A/B in Taiwan [33]. Wong *et al.* reported that 45% of MRSA ST9 strains isolated from pig carcasses in Hong Kong harbored the *qacA*/*B* antiseptic-resistance gene [10]. These differences may be due to geographical specificity, and different origins of MRSA such as animals and humans may have unique prevalence patterns for antiseptic resistance gene carried. The MIC values of Taiwan LA-MRSA harboring *qacEΔ1* for benzethonium chloride and benzalkonium chloride were higher than those reported in other studies (*i.e.*, means of 4–5 μg mL^−1^ in porcine MRSA ST9 in Hong Kong and 0.5–16 μg mL^−1^ in human MRSA patient isolates in Taiwan) [10,16]. The high level of antiseptic-resistance may also be due to the selective pressure imposed by the use of various disinfectants [34]. Because quaternary ammonium disinfectants, such as benzethonium chloride and benzalkonium chloride, are commonly used in livestock practices in Taiwan, these substances may lead to the increased frequency of antiseptic-resistance genes in LA-MRSA isolates. The findings suggested that high MIC of LA-MRSA isolates to antiseptics may contribute to high level of resistance to disinfectants in the LA-MRSA clone found in the swine population, and that this resistance may be transferred to the community setting via meat and related products. The potential public health threat of LA-MRSA ST9 carrying *qacEΔ1* should not be ignored. As a result of the high frequencies of *intI1* and *qacEΔ1* in swine slaughterhouse wastewater, our results implied that the class 1 integrons in wastewater may pose a possible critical platform for spreading of *qacEΔ1* and that wastewater might play an important role in the transfer of *qacEΔ1* to LA-MRSA. Interestingly, although the *intI1* and *qacEΔ1* genes have been commonly detected in municipal wastewater, only one municipal MRSA isolate (3.7%) in the present study harbored the *qacEΔ1* genes. Additional studies are needed to determine whether LA-MRSA tends to harbor the *qacEΔ1* genes more frequently than does human MRSA.

## 5. Conclusions

This is the first study, to our knowledge, to examine the comparative abundances of *intI1* and *qacEΔ1* genes in municipal and swine slaughterhouse wastewater. The results suggest that these two sources of wastewater may become potential hotspots for the development and dissemination of class 1 integrons and antiseptic-resistance genes. Antiseptic-resistant LA-MRSA strains were detected in swine slaughterhouse wastewaters, indicating that such wastewaters may be one of the potential sources of MRSA infection in humans and animals.
